# A review on phytochemical and pharmacological investigations of miswak (*Salvadora persica* Linn)

**DOI:** 10.4103/0975-7406.76488

**Published:** 2011

**Authors:** Jamal Akhtar, Khalid M. Siddique, Salma Bi, Mohd Mujeeb

**Affiliations:** Central Council for Research in Unani Medicine, Department of Pharmacognosy and Phytochemistry, Faculty of Pharmacy, Jamia Hamdard, New Delhi, India; 1 Bioactive Natural Product Lab, Department of Pharmacognosy and Phytochemistry, Faculty of Pharmacy, Jamia Hamdard, New Delhi, India

**Keywords:** Miswak, oral hygiene, salvadora

## Abstract

The miswak is a natural toothbrush made from the twigs of the *Salvadora persica* (Salvadoraceae). Its use predates the inception of Islam and is frequently advocated in the Hadith (the traditions relating to the life of Prophet Muhammad^PBUH^). In addition to strengthening the gums, it prevents tooth decay, eliminating toothaches and halt further increase in decay that has already set in. It creates a fragrance in the mouth, eliminates bad odor, improves the sense of taste, and causes the teeth to glow and shine. The other parts of the tree have therapeutic values as corrective, deobstruent, liver tonic, diuretic, analgesic, anthelmintic, astringent, lithontriptic, carminative, diuretic, aphrodisiac, and stomachic. The present review is therefore an effort to give detailed survey of the literature on phytochemistry and pharmacological activities of miswak.

The *Salvadora persica* (Salvadoraceae) tree drives its Persian name, *Darakht-e-miswak* or tooth brush tree, from the fact that wood is much employed for the manufacturers of tooth brush. It is a large much-branched, evergreen shrub or a tree, found in the dry and arid regions of India, and on saline lands and in coastal regions just above the high water mark. Bark is dull grey or grey-white, deeply cracked, and leaves are variable in shape – elliptic-ovate or ovate-lanceolate – somewhat fleshy. Flowers are pedicellate, greenish-white or greenish-yellow in lax panicles, drupes are globose or round, smooth, red when ripe. The trees readily regenerate from seeds and coppice well [Figures 1–3].[1]

**Figure 1:**
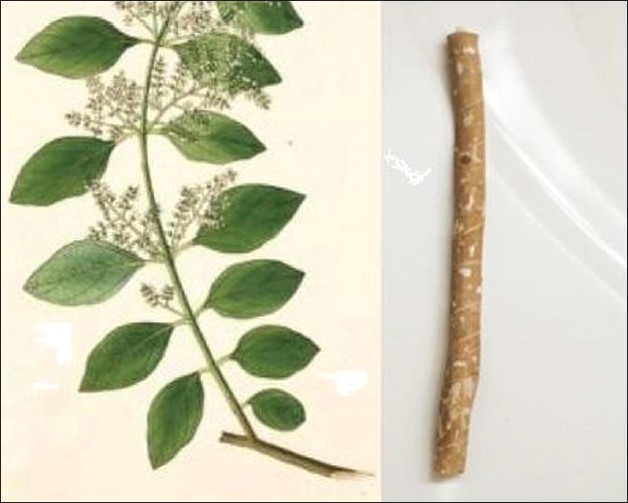
Miswak (*Salvadora persica*) leaves and root

**Figure 2:**
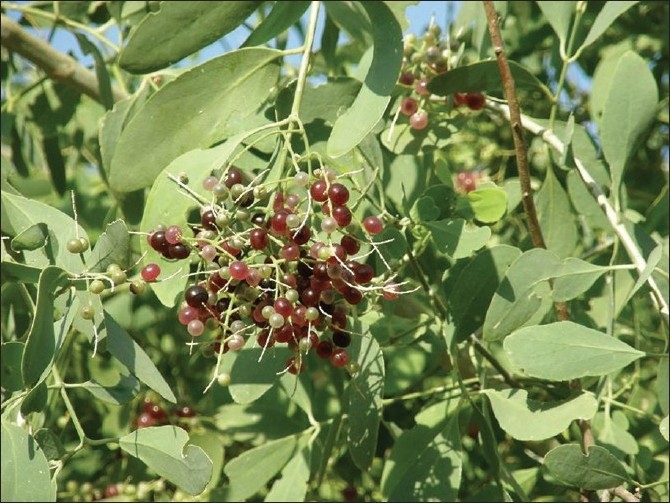
Miswak (*Salvadora persica*) stem branches with flowers and fruits

**Figure 3:**
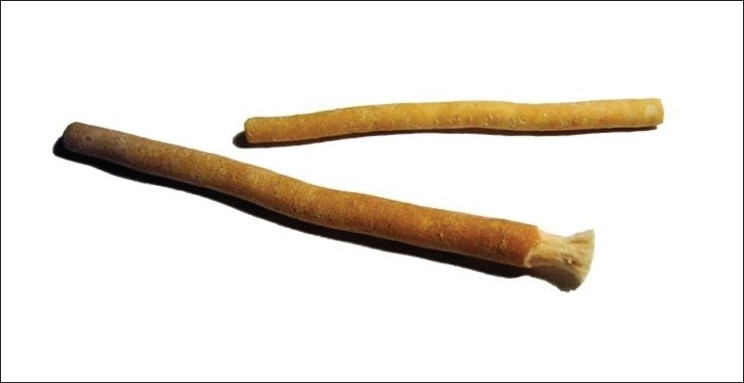
Miswak (*Salvadora persica*) tooth brush

Leaves are eaten as a vegetable in eastern tropical Africa and are used in the preparation of a sauce, and tender shoots and leaves are eaten as salad. Fruits are sweet and edible. A fermented drink is reported to be made from the leaves.[1]

Fresh root bark is used as a vesicant and is employed as an ingredient of snuff. A paste of roots is applied as a substitute of mustard plaster and its decoction is used against gonorrhea and vesical catarrh. The extract of root is said to relieve the pain due to spleen troubles. A decoction of bark is used as a tonic and stimulant in low fevers and as an emmenagogue. Stem bark is used as an ascarifuge and for gastric troubles.[1]

Leaves are bitter and possess antiscorbutic,[1] corrective, deobstruent, liver tonic, diuretic, analgesic, anthelmintic,[2] and astringent[1–2] properties and used in piles, scabies, leucoderma, strengthen the teeth, ozoena, and other nose troubles.[2] A decoction of leaves is used in asthma and cough, and a poultice made out of them is applied to painful piles and tumors. Leaves are also used as an external application in rheumatism. Dried leaves in small doses are given with copious amount of water for the treatment of flatulent dyspepsia.[1]

Fruits possess lithontriptic,[1] carminative,[1–2] diuretic,[1–2] aphrodisiac, alexiteric, appetizer, and stomachic[1–2] properties and are used in biliousness,[1–2] and rheumatism.[1] Seeds have a bitter, sharp taste. They are considered as purgative, diuretic,[1–2] and liver tonic.[2] Seeds oil is applied on the skin in rheumatism.[1]

## Phytochemical Profile

A phytochemical investigation of stems from *S. persica* by Khalil resulted in the first isolation of four benzylamides from a natural source. The isolated compounds were identified as butanediamide, *N*^1^, *N*^4^ -bis(phenylmethyl)-2(S)-hydroxy-butanediamide (I), *N*-benzyl-2-phenylacetamide (II), *N*-benzylbenzamide (III), and benzylurea (IV).[3]

Phytochemical investigation revealed that it contains oleic, linolic, and stearic acids. Among the compounds identified are esters of fatty acids and of aromatic acids, and some terpenoids.[4]

The major components from the essential oil of the toothbrush tree *S. persica* stem have been identified as 1,8-cineole (eucalyptol) (46%), α-caryophellene (13.4%), β-pinene (6.3%), and 9-epi-(E)-caryophellene.[5]

GC-MS analysis of the volatile oil extracted from *S. persica* leaves revealed benzyl nitrile, eugenol, thymol, isothymol, eucalyptol, isoterpinolene, and β-caryophyllene as important constituents.[6]

Sticks from *S. persica* have been analyzed for their soluble and total content of fluoride, calcium, phosphorus, and silica. There was a substantial amount of silica in the ashes of miswak.[7]

The aqueous extract of stem and root of *S. persica* L. has also been investigated for some antimicrobial anionic components by using capillary electrophoresis techniques. It was reported that the root and stem extracts contain sulfate chloride, thiocynate, and nitrate.[8]

Physicochemical analysis of air-dried root bark of *S. persica* was carried out by Bhandari in 1990. He found that it contains 27.1% ash, consisting of considerable amounts of salts, mostly as chlorides. The drug has large amount of alkloidal constituents (including trimethyl amine and unidentified alkaloids), small amount of resin and coloring matter, and traces of tannins and saponins. Higher concentration of fluoride and silica, sulfur, vitamin C, small amount of flavonoids and sterols were also reported.[9–11]

Three lignin glycosides have been reported from the stem of *S. persica*.[12] The flavonoids rutin and quercetin were detected in the stem of *S. persica*.[13] Salvadourea has been reported in the root of *S. persica*.[14] Benzylisothiocynate was also isolated from the root.[15] Salvadoricine, a new indole alkaloid, was reported in the leaves of *S. persica*.[16]

## Pharmacological Profile

### Antimicrobial activities

Aqueous and methanol extracts of *S. persica* were investigated by Firas *et al*. for its antimicrobial activities against seven isolated oral pathogens – *Staphylococcus aureus, Streptococcus mutans, Strep. faecalis, Strep. pyogenis, Lactobacillus acidophilus, Pseudomonas aeruginosa, and Candida albicans* – using disc diffusion and microwell dilution assays. According to both antimicrobial assays, the aqueous extract inhibited all isolated microorganisms, especially the *Streptococcus* spp., and was more efficient than the methanol extract, which was resisted by *L. acidophilus* and *P. aeruginosa.* The strongest antibacterial activity was observed using the aqueous extract against *Strep. faecalis* (zone of inhibition: 22.3 mm; MIC: 0.781 mg/ml). Both extracts had equal antifungal activity against *C. albicans* based on the turbidity test (MIC: 6.25 mg/ml).[17]

*In vitro* antibacterial effect of miswak pieces without extraction has been found most pronounced on *P. gingivalis, A. actinomycetemcomitans,* and *H. influenzae*, less on *Strep. mutans*, and least on *L. acidophilus*. Miswak embedded in agar, or suspended above the agar plate, had strong antibacterial effects against all bacteria tested. The antibacterial effect of suspended miswak pieces suggested the presence of volatile active antibacterial compounds.[18]

Miswak (*S. persica*) extract inhibits the growth of some dental plaque bacteria, and antibacterial effect of the herbal toothpaste was significantly greater than that of the placebo.[19]

Aqueous extracts of miswak and derum enhance the growth of fibroblasts and inhibit the growth of cariogenic bacteria, with the derum extract showing greater activity than miswak.[20]

Antimicrobial activity of eight commercially available mouthrinses and 50% miswak extract against seven microorganisms was compared by Almas and Ahmad in 2005. Corsodyl, Alprox, Oral-B advantage, Florosept, Sensodyne, Aquafresh Mint, Betadine, and Emoform mouthrinses were used, while 50% aqueous extract of miswak (*S. persica*) was used against *Strep. faecalis, Strep. pyogenis, Strep. mutans, C. albicans, Staph. aureus*, and *Staph. epidermidis.* Mouthrinses containing chlorhexidine (CHX) had maximum antibacterial activity, while cetylpyridinium chloride mouthrinses had moderate, and miswak extract had low antibacterial activity.[21]

Antimicrobial activity of Neem and Arak chewing stick’s aqueous extracts at various concentrations was compared by some research workers. Data suggested that both chewing stick extracts was effective at 50% concentration on *Strep. mutans* and *Strep. faecalis*. Arak extract was more effective at lower concentrations for *Strep. faecalis.*[22]

### Cytotoxic activity

The cytotoxic activity of *S. persica* and CHX *was* evaluated by Rajabalian *et al*. in 2009. The results indicated that both persica and CHX mouthwashes were toxic to macrophage, epithelial, fibroblast, and osteoblast cells in a concentration-dependent manner.[23]

### Tick-repellent properties

The *S. persica, Pistacia,* and *Juniperus phoenicea* were evaluated by Garboui *et al*. using host-seeking nymphs of *Ixodes ricinus* in the laboratory for tick-repellent effects of the essential oils. Significant tick-repellent effects were observed for the oils of all three species, but the duration of action was short.[24]

### Antidental caries potential

The efficacy of natural toothbrush or miswak in the prevention of dental caries has been investigated and compared with the efficacy of ordinary toothbrush and toothpaste. The data collected at the end of the study showed that the risk of dental caries for each tooth in the control group was 9.35 times more than the case group.[25]

Less than two-thirds of the sampled adults followed the recommended toothbrushing frequency of twice daily or more, and the majority of subjects did not have a preventive dental visit in the previous 6 months. Furthermore, most subjects reported multiple oral health problems that are mostly preventable through adequate oral hygiene habits and regular preventive dental visits.[26]

Rinsing with miswak extract (*S. persica*) stimulated parotid gland secretion and raised the plaque pH, suggesting a potential role in caries prevention.[27]

### Anti-inflammatory and analgesic potential

Mansour *et al.* evaluated the extract of root and branches of *S. persica* for analgesic activity in mice. It was found that the drug possesses a relatively moderate analgesic effect which might be due to interaction with the central and/or peripheral opiate system.[28]

The extract of stem of *S. persica* was reported to possess anti-inflammatory activity.[29]

### ACE-inhibiting ability

*In vitro* screening has reported that *S. persica* possesses high ACE-inhibiting ability.[30]

### Antiplasmodial activity

Nineteen plant species, used traditionally in Sudan against malaria and similar tropical diseases, were evaluated for pharmacological activity by Ali *et al.* Different extracts of *S. persica* against *P. falciparum* NF54 strain were found to possess antiplasmodial activity.[31]

### Antiplaque activity

It has been observed that miswak was as effective as a toothbrush for reducing plaque on buccal teeth surfaces both experimentally and clinically.[32]

The water extract (10%) of *S. persica* is an effective antimicrobial agent when utilized clinically as an irrigant in the endodontic treatment of teeth with necrotic pulps.[33] Another study compared the oral health efficacy of persica mouthwash (containing an extract of *S. persica*) with that of a placebo. The study showed that use of persica mouthwash improves gingival health and lower carriage rate of cariogenic bacteria when compared with the pretreatment values. Neither the persica nor the placebo reduced the accumulation of dental plaque.[34]

Scientific evaluation of use of miswak revealed that it is at least as effective as toothbrushing for reducing plaque and gingivitis and that the antimicrobial effect of *S. persica* is beneficial for prevention/treatment of periodontal disease.[35]

A clinical study was conducted using patients’ saliva and measuring the effect of miswak (chewing stick), miswak extract, toothbrush, and normal saline on mutans and lactobacilli by Almas and Al-Zeid. The results showed that there was a marked reduction in *Strep. mutans* among all groups. When the groups were compared, the reduction in *Strep. mutans* was significantly greater using miswak in comparison to toothbrushing and there was no significant difference for lactobacilli reduction. The investigators concluded that miswak has an immediate antimicrobial effect. *Strep. mutans* were more susceptible to miswak antimicrobial activity than lactobacilli.[36] Persica mouthwash significantly lowers the gingival index, plaque index, and bleeding index in case group without any reported side effects.[37]

### Effects on fertility

Darmani *et al*. investigated the effects of an extract of miswak for 30 days on the reproductive system of the mouse. The results showed that the exposure to miswak extract did not have much effect on female mouse fertility, although it caused a significant decrease in the relative weights of the ovary and an increase in uterine weights. Exposure of male mice to miswak extract resulted in a 72% reduction in pregnancies in untreated females impregnated by test males. The relative weights of the testes and preputial glands were significantly increased and that of the seminal vesicles was significantly decreased in test males.[38]

### Anticonvulsant and sedative potential

The effect of *S. persica* stem extracts on the potentiation of sodium pentobarbital activity and on generalized tonic-clonic seizure produced by pentylenetetrazol (PTZ) on the rats was observed by Monforte *et al*. The extracts of *S. persica* extended sleeping time and decreased induction time induced by sodium pentobarbital; in addition it showed protection against pentylenetetrazol-induced convulsion by increasing the latency period and diminishing the death rate.[39]

### Antiulcer activity

The antiulcer activity of decoction of *S. persica* has been reported against ASA-induced ulcer in rats. The ulcer index significantly decreased after the treatment with a lyophilized decoction of *S. persica* (500 mg/kg, os), once daily for 7 days, with respect to controls. Moreover, *S. persica* decoction possesses significant anti-inflammatory activity.[40] The other study was designed to confirm the antiulcer activity of *S. persica* decoction using optical microscopy. The elements of gastric mucosa tended to be reestablished normally in treated rats.[41]

### Removal of smear layer and occlusion

Soaking the healthy and periodontally diseased root dentine in miswak extract resulted in partial removal of smear layer, and occlusion of dentinal tubules was observed in dentine specimens brushed with miswak solution.[42] *S. persica* contains potential antimicrobial anionic components, and the capillary electrophoresis is a convenient method for their identification and quantification.[43]

### Antihyperlipidemic activity

The effects of prolonged administration of a lyophilized stem decoction of *S. persica* have also been investigated in diet-induced rat hypercholesterolemia. The results showed that the *S. persica* decoction significantly lowered cholesterol and LDL plasma levels in rats.[44]

### Antimycotic potential

Al-Bagieh *et al.* showed that miswak extract at a concentration of 15% and above has a fungistatic effect for up to 48 hours. The antimycotic effect was probably due to one or more of the root contents which included chlorine, trimethylamine, and alkaloid resin, and sulfur compounds.[45]

### Locomotor activity

Mice injected with *S. persica* extracts showed significantly low exploratory locomotor activity.[46]

### Hypoglycemic activity

Trovato *et al*. observed significant hypoglycemic activity of *S. persica* in rats.[47]

## Clinical Study

The effects of CHX and persica mouth rinses on periodontal status of patients undergoing fixed orthodontic were compared by Poosti *et al*. Gingival index had a significant reduction in all groups after prescribing mouth rinses but this reduction was not significant between groups. Mean pocket depth in CHX group and gingival bleeding index in persica group had significant reduction. Plaque index did not show significant reduction in any of the groups.[48]

## Conclusion

It is concluded that miswak (*S. persica*) reduces the microbial count in different groups and improves the oral health. The extract possesses antibacterial and antiplaque property and it can be used effectively as a natural tool for teeth cleansing and as a natural analgesic for the disturbing toothache. The drug is also reported to possess anti-inflammatory, anticonvulsant, sedative, antiulcer, hypolipidemic, and hypoglycemic activities. The present review showed that it is useful in a number of diseases. Therefore it is imperative that more clinical and pharmacological studies should be conducted to investigate unexploited potential of this plant. The research workers have isolated many phytoconstituents from the plant. Nevertheless further investigations are required to isolate and purify novel pharmacologically active and industrially important compounds.
